# Supporting Teens with Chronic Pain to Obtain High School Credits: Chronic Pain 35 in Alberta

**DOI:** 10.3390/children3040031

**Published:** 2016-11-19

**Authors:** Kathy Reid, Mark Simmonds, Michelle Verrier, Bruce Dick

**Affiliations:** 1 Stollery Children’s Hospital, Alberta Health Services, Edmonton, AB T6G 2B7, Canada; mark.simmonds@ualberta.ca (M.S.); bruce.dick@ualberta.ca (B.D.); 2 Department of Anaesthesiology and Pain Medicine, University of Alberta, Edmonton, AB T6G 2R3, Canada; mrheault@ualberta.ca

**Keywords:** chronic pain, adolescent, school, cognitive-behavioural therapy

## Abstract

Chronic pain is a significant problem in children and teens, and adolescents with chronic pain often struggle to attend school on a regular basis. We present in this article a novel program we developed that integrates attendance at a group cognitive-behavioural chronic pain self-management program with earning high school credits. We collaborated with Alberta Education in the development of this course, Chronic Pain 35. Adolescents who choose to enroll are invited to demonstrate their scientific knowledge related to pain, understanding of and engagement with treatment homework, and demonstrate their creativity by completing a project, which demonstrates at least one concept. Integrating Chronic Pain 35 into an adolescent’s academic achievements is a creative strategy that facilitates the engagement of adolescents in learning and adopting pain coping techniques. It also helps teens to advocate for themselves in the school environment and improve their parents’ and teachers’ understanding of adolescent chronic pain. This is one of the first successful collaborations between a pediatric health program and provincial education leaders, aimed at integrating learning and obtaining school credit for learning about and engaging in health self-management for teens. The authors hope this paper serves as an effective reference model for any future collaborating programs aimed at supporting teens with chronic pain to obtain high school credits.

## 1. Introduction

Chronic pain is a significant problem in the pediatric population. Chronic pain is defined as any prolonged pain that lasts longer than the expected healing time or any recurrent pain that occurs at least three times during a three-month period [1,2]. Common pain diagnoses in children and adolescents include headaches, chronic abdominal pain, back pain, and musculoskeletal pain [3]. The pain may result from a disease, injury, a trauma, or surgery. For a significant number of children, there are no identifiable causes for the reported pain. Increasingly, this type of pain is discussed as a result of neurobiological changes that result in pain syndromes including pain centralization and pain amplification [4,5]. Population studies demonstrate that 20%–35% of children and adolescents report symptoms meeting research definitions of chronic pain [1,2,6]. It is estimated that between 5%–8% of children with chronic pain will develop significant pain-related disability [7,8]. This pain-related disability is known to affect children’s functioning including their ability to attend school on a regular basis [9]. These children often require an interdisciplinary team to help manage chronic pain and pain-related disability.

Research has demonstrated that adolescents with chronic pain report being less socially developed than their peers [10] and that they may avoid social situations with peers [11]. Lower social functioning can contribute to school impairment, and therefore having children socially connected to their peers can limit the detrimental effects on school functioning [12].

As mentioned, evidence demonstrates that children and adolescents with chronic pain often struggle to attend school on a regular basis [9]. In one study, 44% of students with chronic pain missed at least 25% of school days, and 20% missed more than one half of school days [13]. In addition, 44% of the students reported a decline in grades. A study comparing school absences of children with functional abdominal pain (FAP), inflammatory bowel disease (IBD), and healthy controls found that children with both IBD and FAP missed significantly more school than healthy controls [14]. In the Pediatric Pain Program at the Stollery Children’s Hospital (Edmonton, AB, Canada), our records suggest that a majority of individuals presenting with chronic pain miss at least one half to two days per week on average, and approximately 15%–20% of adolescents are either no longer attending school on a regular basis or have stopped attending in person and complete their studies in a correspondence program. In addition, chronic pain is shown to affect children’s cognitive function, but more research is required to determine the long-term effects of this cognitive dysfunction on the developmental trajectory of young people with chronic pain [15].

Given the prevalence of chronic pain, and the magnitude of its effects on adolescents’ social development and school functioning, it is critical that teens be provided with the opportunity to continue to learn. Additionally, young people in our program report that it is important to their mental health and self-concept to make progress in their educational programming and pursuit of their school and life goals, despite experiencing ongoing pain. It is also important to maintain contact with a peer group, in order to support normal social adolescent development.

## 2. Managing Chronic Pain: Biopsychosocial Approach

Adolescents with chronic pain benefit from multimodal approaches to improve their function and return to regular activities, including school. Interventions that include the 3Ps—physical, psychological and pharmacological approaches—are more likely to be successful than single interventions. One of the most effective treatments for chronic pain is cognitive-behavioural therapy (CBT), which has been shown to be effective in managing headache, recurrent and functional abdominal pain in children [16], sickle cell disease [17], and juvenile fibromyalgia [18]. CBT strategies for chronic pain management address pain education, and teach active coping strategies such as relaxation and imagery, stress management, cognitive restructuring, goal setting, and relapse prevention [19]. Treatment approaches that enhance self-efficacy, or increase confidence to function despite pain, have been suggested as helpful for youth with headaches [20,21].

A group intervention for adolescents with chronic pain (*n* = 40) and their parents, called “Coping with Pain School” in Boston, MA, USA, by Logan and Simons [22] provided either four sessions of CBT or a day long CBT session aimed at improving school function. The study found the program was feasible, families who participated were satisfied with the program and school attendance improved after completion of the sessions; however, enrollment challenges were a significant problem identified by the authors in the study. They recommended the need to develop treatments focusing on school functioning and involving school personnel in future studies.

## 3. The Stollery Experience

The Pediatric Chronic Pain Program at the Stollery Children’s Hospital opened in 2008, to provide treatment for children and adolescents with chronic, difficult to manage pain. The large geographical catchment area includes northern Alberta, the Northwest Territories, and North Eastern British Columbia. Children from Saskatchewan and Manitoba have also been included. All patients must be referred to the clinic by a physician or nurse practitioner and must meet the clinic’s referral criteria (Table 1).

The family attends an intake appointment in person with the full interdisciplinary team to share their pain experience. The team consists of a chronic pain physician, a nurse practitioner, a physiotherapist and a psychologist. The initial appointment takes up to two hours to complete a comprehensive physical and psychosocial history, physical examination and development of a treatment plan with input from the child and family. For many of the children, participation in group cognitive behavioural therapy becomes part of the treatment plan. This program, called Pain 101, consists of 10 sessions, provided jointly by the psychologist and nurse practitioner. Pain 101 is a treatment program provided by our clinic to children 12–18 years old. Although the treatment is offered for a broad range of ages, due to the careful screening process that takes place prior to Pain 101 admission, we have yet to experience significant issues associated with developmental differences in participant responses.

Participants are invited to attend the sessions in person or by telehealth (secure videoconferencing). Given the large catchment area of our program, telehealth is offered to children who reside outside of the city, which makes attending the program much more feasible, especially to children whose school has access to this secure videoconference service. Pain 101 classes take place in the afternoon to best accommodate high school students who can set their schedule to avoid missing core classes. Group sizes for the class range from 5–15 participants for each 10-session block. Teens who participate are expected to attend all sessions and complete the homework as outlined. In addition to the classes for teens, sessions for parents are offered and provided by the nurse practitioner. The purpose of these sessions is to help parents understand the science of chronic pain, factors that exacerbate pain, and self-management techniques aimed at promoting increased function, mental health, coping, self-efficacy, mindfulness, acceptance, and self-compassion. Attendance for these sessions is encouraged but not required for the program, and consequently attendance of the parent session varies. However, since implementing a parallel course, Chronic Pain 35 (see Section 4), we have noted an increase in the number of parents who attend, as well as requests for additional sessions to be held. For parents, sessions aim to teach them appropriate developmental expectations, strategies to facilitate children’s self-management and parent coping all with a global aim help their child function despite pain. It is important that parental behaviours, especially protectiveness [23] and parent pain–related fear [24], be addressed in order to facilitate the return to school for the small but significant number of students who miss extensive amounts of school.

### 3.1. Pain 101 Session Content

#### 3.1.1. Pain Education

**Figure 1:**
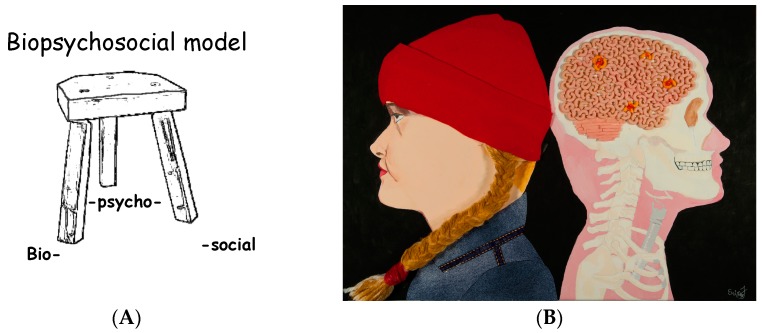
(**A**) Biopsychosocial model—the example of a three-legged stool is often used to discuss this topic with the participants, focusing on the concept that all three legs are needed to be able to sit on the stool; (**B**) this image, created by a student of the Pain 101 course entitled “Untitled”, is often used to assist in the explanation of the biopsychosocial model, in order to explain pain locations in the brain to participants.

The biopsychosocial model of chronic pain is introduced (Figure 1), and this model is reviewed throughout Pain 101. Pain pathways in the body are reviewed, including how pain messages are sent from the periphery and received by the brain. Participants learn how chronic pain differs from acute pain. The Gate Control Theory of Pain is introduced and up-to-date scientific information on our understanding of this theory is discussed. For homework, participants are asked to complete specific questionnaires.

#### 3.1.2. Tension and Relaxation

Participants learn how tension opens pain gates and thereby increases pain and how relaxation strategies have the potential to modify the pain experience. Relaxation exercises including progressive muscle relaxation, guided imagery and diaphragmatic breathing are taught and practiced with the group. For homework, participants are asked to complete relaxation diaries and practice the techniques daily.

#### 3.1.3. Pacing

Participants learn how activity avoidance and activity cycling can open pain gates and lead to disability in chronic pain. Methods to break this downward cycle are reviewed. Participants are expected to complete activity goals for themselves. The goals are broken down from long-term goals to intermediate goals to mini goals. Activity tolerance times are calculated, and then, from this time, baseline activity times are calculated. Participants are then expected to meet their baseline activity goals on a daily basis. For homework, participants are asked to develop a specific, measurable, achievable, relevant and time dependent (S.M.A.R.T.) goal and determine tolerance and baseline for individual activities.

#### 3.1.4. Dealing with Negative Thoughts

Participants learn how living with chronic pain can be associated with negative thoughts and negative moods, including sadness or being overwhelmed, and may even contribute to depression or unhealthy, negative coping behaviours. Methods to challenge these negative thoughts are reviewed including active and positive coping strategies, reframing thoughts, recognition of feelings and challenging unhelpful thoughts. For homework, participants are asked to complete the daily thought diary.

#### 3.1.5. Mindfulness

Participants review the theory of mindfulness-based stress reduction. Practice activities for mindfulness are completed in class. Participants are encouraged to practice various daily activities in a mindful way, such as taking the bus, exercising, and completing chores. For homework, participants are asked to complete one mindfulness activity daily.

#### 3.1.6. Sleep and Nutrition

Participants review the importance of sleep in managing chronic pain and closing pain gates. Healthy sleep practice strategies that support restorative sleep are reviewed, including cognitive, behavioural, and environment modification sleep practices. Dietary habits that affect pain and sleep, including caffeine and energy drinks are also reviewed. For homework, participants are asked to perform a practical application of sleep hygiene strategies and a minimum of one dietary change.

#### 3.1.7. Stress and Anxiety

In this session, we discuss how stress and anxiety lead to biological responses that affect pain gates, mood, and sleep, thereby exacerbating pain. Participants review stressors, such as school, homework, dealing with others, and chores. Fear of pain and how that fear contributes to disability are also reviewed. Recognizing the physical effects of stress and anxiety, including physical reactions (fight or flight response) and emotional reactions are discussed. Additionally, methods to decrease stress and anxiety are reviewed, such as recognition and coping strategies that include relaxation and pacing activities. For homework, participants are asked to recognize personal stressors and find coping strategies.

#### 3.1.8. Communication

Pain behaviours and communication are reviewed. Participants learn about both verbal and non-verbal communication messages and discuss how the messages may be interpreted by others. This session includes a video demonstrating how society sees what they are conditioned to see. Discussion takes place regarding how strategies can be practically applied to teach family, friends, and others about pain. For homework, participants are asked to complete a communication diary.

#### 3.1.9. Setback Planning (Relapse Prevention)

Participants learn how to develop their own personal setback plan to be utilized whenever they have a flare in their pain. This plan includes recognizing the flare early, implementing the various strategies reviewed through the course—relaxation, pacing, thoughts, sleep, stress, and communication. For homework, participants are asked to complete their setback plan worksheet.

#### 3.1.10. Life with Pain

**Figure 2:**
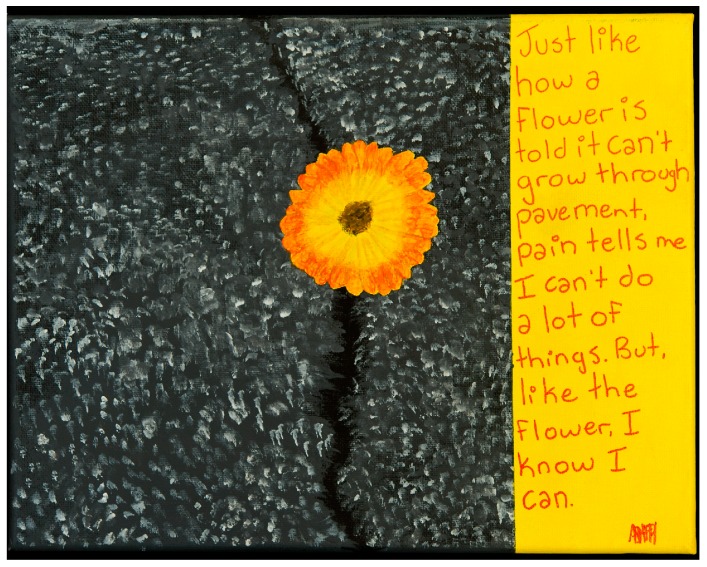
“A flower can grow” created by a Pain 101 participant, A.F.H.

This final session includes strategies to live life to the fullest despite having pain. Concrete methods include simplifying activities of daily living such as school work and activities, chores, work, social activities, and other activities of daily living. We review how strategies that have previously been covered in Pain 101 can be applied to each of these life activities, structuring activities to allow for breaks while still meeting goals. Individual setback plans are reviewed and participants are asked to provide a copy of their set-back plan to the pain clinic staff. For homework, participants are asked to submit the written set-back plan, along with submission of their Chronic Pain 35 project for those who are registered in the parallel course. An example of participant work from this session can be seen in Figure 2.

### 3.2. Participant Measures

All participants who attend Pain 101 are requested to complete questionnaires (Table 2) as per the Pediatric Initiative on Methods, Measurement, and Pain Assessment in Clinical Trials (PedIMMPACT) recommendations [25]. These data are used for the establishment of a clinical baseline, monitoring, and, for those who consent, research. The two primary findings from our analyses that have been continually replicated are significant reductions in pain-related disability (*p* < 0.001) and anxiety (*p* < 0.005).

### 3.3. Session Completion

The questionnaire measurements are completed prior to the start of Pain 101, at the end of Pain 101 (three months later), and again at the six- and 12-month post-group completion milestones, and then annually at the five-year mark, post-group completion. Additionally, we have begun tracking education outcomes and the number of school days missed in comparison to pre- and post-completion of Pain 101.

## 4. Chronic Pain 35

In 2010, our healthcare team from the pediatric chronic pain clinic, along with several teens, started to work on ways to consistently obtain high school credits for completing the program and demonstrating their learning. Prior to that point, some students had been granted high school credit for completing Pain 101, but the number of credits granted and the categorization of those credits was highly variable between students. It was up to each student to advocate for themselves at their local school, with support from our team, to obtain credits. Several students were able to obtain between one and three credits, but we lacked consistency at a local or provincial level. In 2014, we approached Alberta Education, which is the provincial government agency responsible for public education in Alberta. We explained the program that we were providing and shared samples of the projects completed by students for successful credits at their local school. Together with educational consultants at Alberta Education and the Alberta Distance Learning program (a provincial online school), we developed the course syllabus. This course syllabus included specific learner outcomes for each of the classes that must be demonstrated for successful completion of the course. Examples of outcomes include: Pain Education—identify two methods by which pain gates in the body are opened; Pacing—develop a S.M.A.R.T. goal for a physical activity and demonstrate following through on that goal; Negative Thoughts—identify at least one cognitive distortion and modify negative thoughts associated with the noted distortion. The course was submitted to the Alberta Education curriculum board, and, in February 2015, it was approved as a locally developed course called Chronic Pain 35. Students who successfully complete the course obtain three credits at the Grade 12 (senior year) level in Alberta. In order to obtain a high school diploma in Alberta, students must complete courses which total 100 credits.

Participants attending Pain 101, enrolled in Grades 10–12 (ages 14–18 years old) are eligible to enroll for the course Chronic Pain 35, in order to earn high school credits. Although junior high school students are allowed to attend Pain 101, there is not a credit program in Alberta for grades 7–9, and therefore the Chronic Pain 35 course is restricted to high school students only. The course is only open to students who are followed in our clinic, as parallel attendance in Pain 101 is required. Not all teens who attend Pain 101 register for Chronic Pain 35, since many teens do not need the credits, live outside of Alberta, or are younger than high school age. Enrollment requires completion of the Alberta Distance Learning (ADL) registration form and written consent of a parent/guardian. Prior to registering, we provide the family with a written outline of the course and completion requirements. Additionally, before registration, teens and their parents choosing to enroll are informed that credits for the course “Chronic Pain 35” will appear on their official transcripts. Any ethical concerns expressed regarding this are discussed with the teen and family directly prior to enrollment. To date, only one student expressed concern over the course name appearing on the transcripts, although the student chose to continue and receive credits.

Students who enroll are required to attend all Pain 101 sessions, submit weekly homework assignments, including completion of relaxation diaries, thought challenging worksheets, development of S.M.A.R.T. goals, and a written setback plan for managing pain flares. Any missed sessions must be made up. Additionally, students must complete a project that demonstrates their learning of at least one concept related to the sessions. Projects have been produced using a variety of media, including written work such as essays, personal reflections and poetry. Other projects have used visual arts, such as collages, story boards, paintings, sculptures, and a host of other genres. A small number of students have composed and produced various forms of music. The course mark is 20% for attendance, participation and homework, and 80% for completion of the project. A certified teacher in the province of Alberta has been assigned to the program and grades all course projects. The teacher attends several sessions to meet the students and answer any questions they may have related to the planned project. She is also available via the online distance learning site to discuss projects with the students.

As of July 2016, 35 students have enrolled in Chronic Pain 35. Of these, 32 students have completed the course and received school credits, since three students completed Pain 101, but chose not to complete the course requirements for Chronic Pain 35. Samples of the participant’s work can be found in the Supplementary Material. Feedback from students thus far has been very positive, and attendance and homework completion consistency have markedly risen since the formalization of Chronic Pain 35. To quote one of the 17-year-old student participants: “Without this class I would be short credits to graduate. I used to miss a few days of school each week, but now I barely miss any.” In addition to obtaining school credits, several students have discussed the importance of having the opportunity to meet and learn with other students who have chronic pain, and to be with others who understand what it is like to live with chronic pain. These students motivate each other to incorporate the strategies into their own lives and help support each other in achieving goals. These students have reported that one of the most important aspects of the group work is the opportunity to continue to meet with other teens who “get them”; who truly understand what it is like to live with chronic pain. As demonstrated by Forgeron [33], the majority of students who attend group CBT for chronic pain self-management are interested in friendships with their class peers for emotional support.

## 5. Discussion

This paper highlights our unique program that incorporates attendance at a group CBT program for chronic pain and the provision of high school credits for the completion of a course that is provided in a health care setting. Our CBT program includes many recommendations from previous research studies that have addressed multiple facets of the chronic pain experience while helping young people return to school. School avoidance and absences arise from multiple personal and psychosocial factors. Addressing these factors in a group setting can help youth return to school and thereby improve school performance. In a study of 349 youth treated in a chronic pain clinic, Khan et al. [34] found that anxiety played an important role in both school attendance and function while at school. Addressing anxiety, enhancing self-confidence and self-efficacy, and assisting teens in developing methods to manage anxiety using CBT, are all pillars of practice that are incorporated into our program. Additionally, in a study of 47 adolescents with chronic headache, Claar et al. [35] suggest that having the health care team address methods to return to school can help decrease the frequency and duration of headaches. In several sessions of our program, teens are taught concrete techniques to return to function, such as pacing and goal setting. Additionally, by receiving school credits for completion, the student is increasingly empowered to continue to attend school.

In a study by Logan and Curran [36], 38 teachers were interviewed about their needs when dealing with an adolescent with chronic pain. The study revealed that teachers needed to further understand chronic pain and how to manage pain-related behaviours. In our program, we offer a specific class in which we discuss ways to communicate and how to ask others to help in managing pain, including school personnel. Teachers are further supported by the Alberta Education Chronic Pain 35 curriculum guide, in which teachers can access the relevant information on our program and learn how it can help students succeed. Therefore, the completion of Chronic Pain 35 has opened communication with teachers and school administrators that may not have otherwise occurred. Having a certified teacher involved in the program, who is available to intervene with the teens at a school level, also serves to increase understanding of chronic pain in schools across the province.

As identified by Boutilier and King [37], school sites have been undervalued in helping children with chronic pain management, along with the inherent additional challenges. They recommended increased interdisciplinary collaboration between schools and health care teams. Our program directly meets this recommendation by working with Alberta Education at a local and provincial level to best meet the needs of our students. The program has been endorsed by both the Alberta provincial Education Minister and Health Minister.

### Future Directions

Given that up to 50% of adolescents with chronic pain miss several school opportunities, days of classes, and social involvement with peers, it is essential that programs providing services to these children incorporate school functioning efforts into their treatment plans. As recommended by Gorodzinsky et al. [38], measurement of school functioning should include the assessment of absenteeism, academic performance, and cognitive function within a developmental framework. Our program will continue to use outcome measures to determine both short-term and long-term results in adolescents who complete Chronic Pain 35, including tracking school absences and school functioning. We will continue to request permission from the students to share their projects which demonstrate their learning. It is hoped that other jurisdictions can work with their educational boards at a local, provincial (or other) level to develop similar programs. Most importantly, we will continue to advocate for students to be able to return to the classroom and continue with their education, and we encourage other programs to contact us if they are interested in modeling our program or learning more about our work. Children are the future of our societies and children with chronic pain require unique support from teams, including the collaboration of health care providers and teachers, to meet their goals of learning, graduating, gaining fulfilling employment, and contributing to society.

## Figures and Tables

**Table 1:** Stollery Chronic Pain Clinic referral criteria.

**Request for Consultation: Referral by Physician/Nurse Practitioner only**	The goal of this service is to treat and care for children 17 years and under who are experiencing chronic, difficult-to-manage pain. Children should always have been seen and assessed first by the appropriate pediatric services prior to being referred to the Pediatric Chronic Pain Clinic.
**Inclusion criteria**	• 0–17 years, 11 months
	• chronic pain of at least three months’ duration
	• chronic pain as primary complaint
	• chronic pain which impacts activities of daily living, school attendance, sleep, quality of life or family functioning

**Table 2:** Questionnaire measurements.

**Pain History and Demographics**	Details including age, school grade, gender and school absences are recorded. Additionally, information on pain experience, chronicity, frequency, location and intensity are entered.
**Visual Analogue Scale**	The child is instructed to illustrate, by drawing a line on a scale from 0–10 (0 = no pain, 10 = worst possible pain), the intensity of pain felt in the past week [26].
**Pediatric Quality of Life Inventory**	Participants are asked to report how their pain has impacted their quality of life, including their activities, feelings, social interactions and school performance. Parents are asked to fill out the parent version of this scale which includes the same questions, but reporting their observations of their child’s quality of life [27].
**Children’s Sleep Habits Questionnaire**	The child is asked 26 questions about own bedtime habits, sleep behaviour and daytime sleepiness. The parent form is constructed of 33 questions on child’s bedtime habits, sleep behaviour, waking during the night, morning waking, and daytime sleepiness [28].
**Functional Disability Index**	Child and parent(s) are asked to report the child’s level of physical trouble or difficulty when performing various tasks [29].
**Tampa Scale of Kinesiophobia for Children**	A self-report form asking the child to identify how they cope with pain. This measure records the child’s somatic focus and activity avoidance [30].
**Revised Child Anxiety and Depression Scale**	This 47-item self-report measure assesses the symptoms of separation anxiety, general anxiety, panic, social phobia, obsession/compulsion, and depression. The child is asked to report the frequency (from ‘never’ to ‘always’) of various worrying and sad thoughts [31].
**The Children’s Chronic Pain Stigma Scale (CCPSS)**	This scale is also currently being developed and validated in our program based on the adult Chronic Pain Stigma Scale [32]. This measure explores perceived stigma from physicians, family members and the general public associated with chronic pain.

## Supplementary Materials

The following figures are available online at http://www.mdpi.com/2227-9067/3/4/31/s1, Figure S1: “Shoes” by B.V.; Figure S2: “Dear Non-Believers” by C.S.; Figure S3: “Pain” by A.S.
