# Supplementary material for: Supporting Teens with Chronic Pain to Obtain High School Credits: Chronic Pain 35 in Alberta
Source: Children (Basel). 2016 Nov 19;3(4):31. doi: 10.3390/children3040031 (PMC5184806; doi:10.3390/children3040031)
Supplement: Supplementary file 1 [file children-03-00031-s001.pdf]

Supplementary Materials: Supporting Teens with Chronic Pain to Obtain High School Credits: Chronic Pain 35 in Alberta

Kathy Reid, Mark Simmonds, Michelle Verrier and Bruce Dick

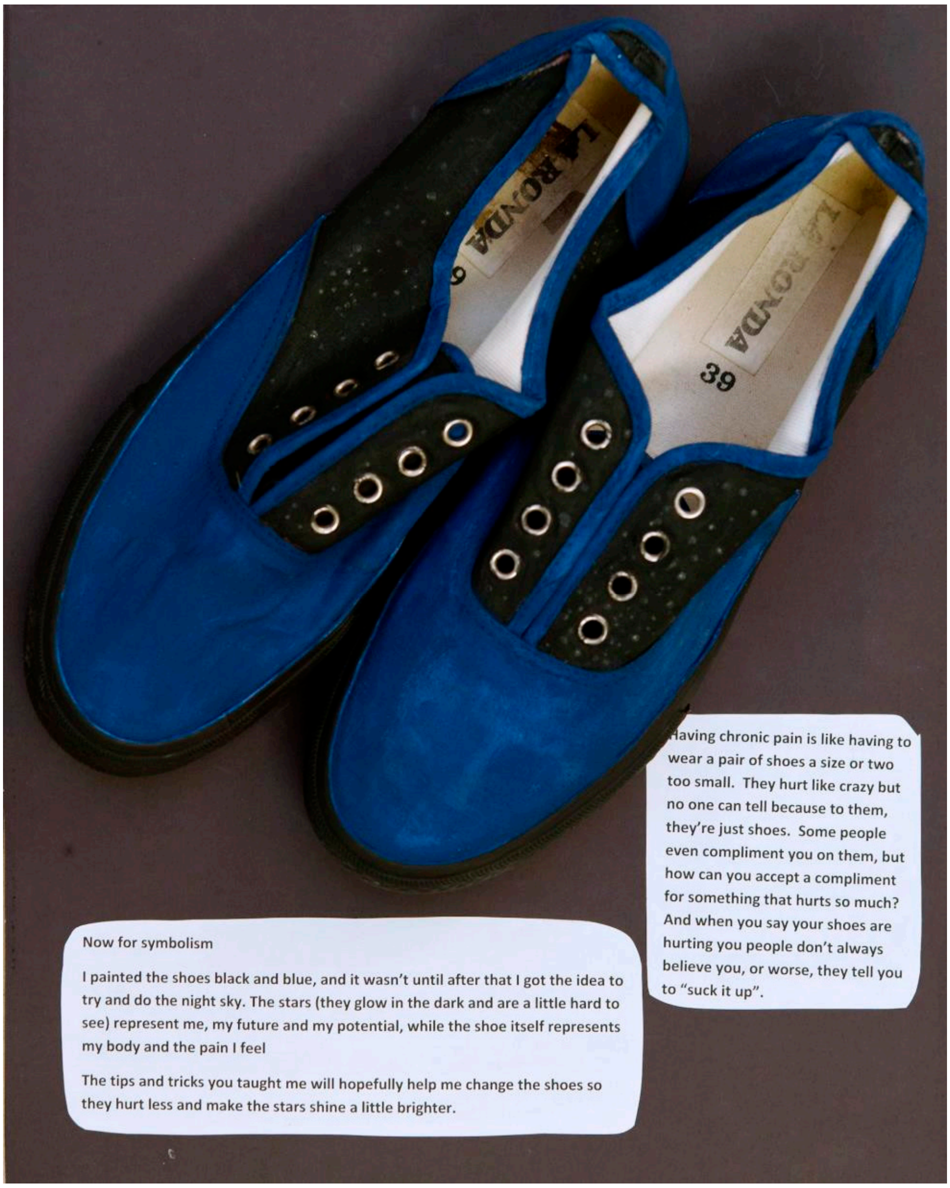

Figure S1. "Shoes" by B.V.

DEAR Non-believers,

So you think my pain IS FAKE do you? You think I like the weekly trips to the hospital, thE CO~~nstant~~ AGony, and the mental and psychological side affeCts it has on my brain?

Because of this pain I can no longer do half of the things I used to liKE AND I CAN'T EVen do some thinGS THAT ARE NECessary to sustain life. Reading this is what a day to day liFE IS LIKE With chronic pain. You cannot focus on the task At hand becaUSE of all of the distractions CaUSE~~D BY YOUR Pain~~. This is what affects me most.

Figure S2. "Dear Non-Believers" by C.S.

Pain

It seems like a never ending event.
An event of pain that just won't end.
What happens on the outside is nothing compared to the pain inside, pure torment.
Your feelings and thoughts are too much to comprehend.
Your thoughts eat away at what little hope you had.
You watch your friends play and watch as people live pain free.
Though you learn to challenge those thoughts and slowly become glad.
Your need for help slowly slips away you no longer feel the need to plea.
Your dark world becomes lighter as you slowly make your way back.
You start returning back to your normal life you can't believe you've made it so far.
You no longer need to numb the pain with that cold unwanted ice pack.
After all you've gone through you've found out who you are.
You're a new person and now you've got it going your way.
You hope for others that they'll find help without any delay.

Figure S3. "Pain" by A.S.
